# Foundation Piles—A New Feature for Concrete 3D Printers

**DOI:** 10.3390/ma14102545

**Published:** 2021-05-13

**Authors:** Marcin Hoffmann, Krzysztof Żarkiewicz, Adam Zieliński, Szymon Skibicki, Łukasz Marchewka

**Affiliations:** 1 Faculty of Mechanical Engineering and Mechatronics, West Pomeranian University of Technology, Piastów 19, 70-310 Szczecin, Poland; lukasz.marchewka@zut.edu.pl; 2 Faculty of Civil and Environmental Engineering, West Pomeranian University of Technology, Piastów 50a, 70-311 Szczecin, Poland

**Keywords:** concrete 3D printing, additive manufacturing, foundation piles, robotic fabrication

## Abstract

Foundation piles that are made by concrete 3D printers constitute a new alternative way of founding buildings constructed using incremental technology. We are currently observing very rapid development of incremental technology for the construction industry. The systems that are used for 3D printing with the application of construction materials make it possible to form permanent formwork for strip foundations, construct load-bearing walls and partition walls, and prefabricate elements, such as stairs, lintels, and ceilings. 3D printing systems do not offer soil reinforcement by making piles. The paper presents the possibility of making concrete foundation piles in laboratory conditions using a concrete 3D printer. The paper shows the tools and procedure for pile pumping. An experiment for measuring pile bearing capacity is described and an example of a pile deployment model under a foundation is described. The results of the tests and analytical calculations have shown that the displacement piles demonstrate less settlement when compared to the analysed shallow foundation. The authors indicate that it is possible to replace the shallow foundation with a series of piles combined with a printed wall without locally widening it. This type of foundation can be used for the foundation of low-rise buildings, such as detached houses. Estimated calculations have shown that the possibility of making foundation piles by a 3D printer will reduce the cost of making foundations by shortening the time of execution of works and reducing the consumption of construction materials.

## 1. Introduction

**Figure 1:**
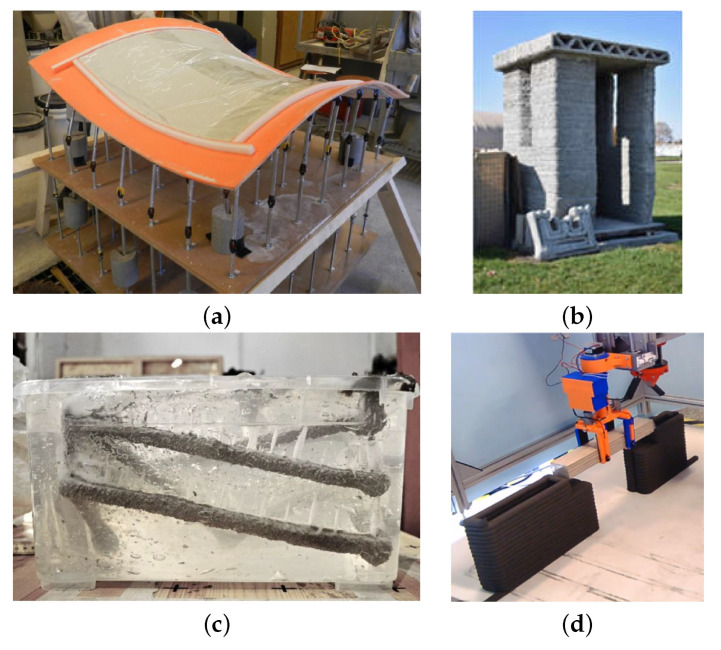
(**a**) Flexible Mould [5], (**b**) Entry Control Point [11], (**c**) material injected into a gel [28], (**d**) use of a gripper for lintel placement [27].

Digital Fabrication with Concrete (DFC) technologies have been developing rapidly in the construction and architecture industry. The work of RILEM Technical Committee 276 presents guidelines according to which DFC technologies can be described and categorised [1,2]. Consideration was given to the materials that were used in the production construction process, the application environment, what the final product is, as well as the processes that are involved in production, their sequence, and implementation. Examples include the Mesh Mold technology, in which the machine operations for the assembly, cutting, bending, and welding processes are numerically controlled while the casting and contour crafting operations are performed manually [3,4]. The Flexible Mould (Figure 1a) method is the manufacture of curved panels on flexible coatings, which, in turn, are applied onto pins that provide the desired shape [5,6].

Additive Manufacturing (AM) is the fastest growing DFC technology in construction. In this technology, three-dimensional structures are formed by the head that is moved in the space according to a pre-designed trajectory and the constructed object is built layer-by-layer [7,8]. This technology is used for the prefabrication of structural elements [9], the manufacture of small architecture [10], and the construction of small utility buildings [11] and residential buildings [12]. It has also found application in the construction of bridges (Figure 1b) [13,14,15] or wind turbine columns [16].

As far as construction is concerned, AM systems are designed for various scopes of work. The primary function of these systems is to lay down successive layers of material to create the desired three-dimensional structure. This makes it possible to form the load-bearing and partition walls of buildings. Printing machines (printers) are based on different kinematic solutions, and Cartesian robots [17,18,19], robotic manipulators [9,20,21], and Delta type manipulators [22,23] are used. Stay-in-place for the construction of support columns [24] or foundations [25] are also printed. Some structural elements of the building cannot be printed on-site or are technologically difficult to print, e.g., elements of stairs, ceilings, lintels, and roofs. These components are manufactured in incremental technology as prefabricated parts and placed at the destination by cranes. In the literature, we can find examples where the printer also plays the role of a crane transporting prefabricated elements during construction (Figure 1d) [26,27].

New printing methods and strategies are being developed, which include six Dof manipulators, new construction materials, or non-standard formwork solutions. This extends the range of construction works that were carried out on-site by the AM system. An example is the use of technical fabric as a lightweight formwork on which successive layers of concrete are applied in stages [29]. Supporting elements in the form of metal frames are used to make roofs [30]. The works [31] present the idea and tests of printing the vault without using supports, following the example of the Nubian vault. ApisCor presented the concept of printing a flat slab by laying concrete trails at the appropriate angle [25]. The articles [28,32] present a method, called Injection 3D Concrete Printing, which is based on the concept that a liquid building material is injected into a gel-type support material with specific rheological properties (Figure 1c). This ensures that the support material maintains the stable position of the construction material. Constructions-3D presented the fabrication of a staircase consisting of 10 modular steps [33]. The work [34] demonstrated the possibility of adjusting printing parameters to print the main structure and the supporting structure using a single type of construction material. Robotic systems enable additional work to be carried out on printed or sprayed fresh concrete. Examples include post-printing material contour crafting [35,36] and the removal of excess material with cutting tools [36]. A work [37] shows the use of a six Dof manipulator to deposit concrete on a textile mesh at different orientation angles.

Another aspect of Digital Fabrication with Concrete technology is designing the right concrete mix. Depending on the type of technology used for 3D printing, the mix that is intended for this purpose must have the appropriate mechanical and rheological properties. In the “Extrusion” printing method, the fresh mix must have rheological properties that will allow it to maintain its shape during the application of successive layers and, at the same time, be characterised by adequate extrudability/pumpability for the assumed period of the so-called “open time” [38]. Controlling the properties of the mix is also important in terms of its load-bearing capacity. The researchers [31] printing vault show that it is important to ensure adequate strength, not only in compression, but also in the complex stress state. In addition, imperfections also have an important influence, which can create additional internal forces in the printed structure [39,40] or affect the issue of stability of the printed component [41,42]. Additionally, the shape of the nozzle outlet or the speed of the print head movement can affect the final shape and bearing capacity of the printed structure [39,43,44,45]. In the case of printing with simultaneous contour crafting of the fresh mix, a better print surface is obtained, which allows for reducing the technological regime of printing [46]. If we consider printing the mix on a textile mesh [37], its flow limit can be much lower than when printed without a support material.

In the techniques described above (“extrusion,” “contour crafting”) the mix is generally designed on a cement binder with various reactive additives, such as fly ash or silica fume [1,38,39,47,48,49]. For technological reasons, mixes usually only contain fine aggregate [38,47,48,49,50,51]. The fine aggregate used is often modified with various micro-fillers to improve the rheological properties [52,53,54,55,56]. The preservation of the required parameters is also achieved by using combinations of chemical admixtures [39,46,47,49,57,58]: accelerating, slowing down, modifying viscosity, or other.

A completely different approach to mix design must be taken when printing in a support material [28,32], where it is important to protect the mix from the adsorption of its components by the absorbent support material by using viscosity modifying admixtures. Printing when using support materials based on the Particle Bed Fusion technique [59,60,61] involves injecting a binder into a specially prepared substrate. In this case, the substrate becomes the construction material after activation with a cement or polymer-cement slurry. This technology requires a substrate made of specially selected aggregate as well as reactive additives and microfillers.

**Figure 2:**
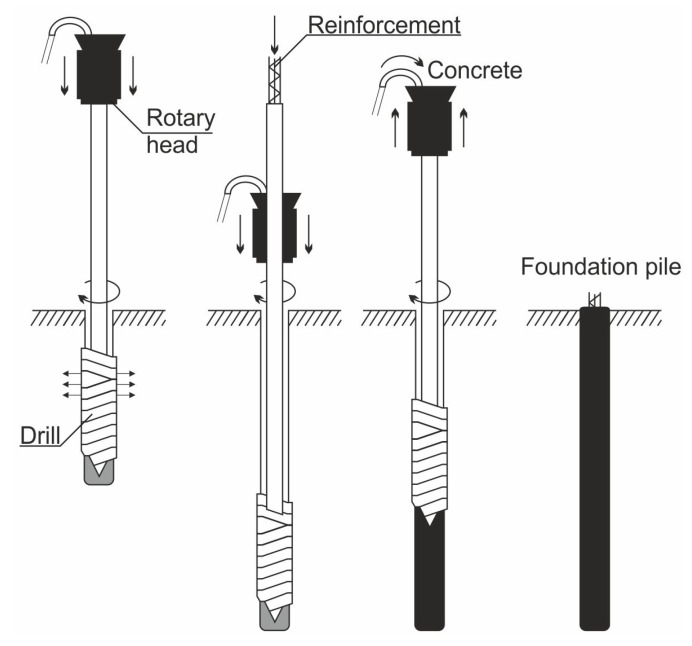
Diagram for execution of a displacement pile based on [63].

The load-bearing capacity of the soil on which the facility is to be erected plays an important role in construction planning. In the case of bearing soils, direct foundation in the form of strip foundations, spot footings, and slabs constitutes the basic type of foundation. These are the structural elements that directly transfer the load from the structure to the soil. In the case of weak-bearing layers, soil strengthening measures such as compaction, injection or soil replacement can often be applied. In the case where weak-bearing layers are deeply layered, the most common method is to use intermediate foundations in the form of wells and piles up to the bearing layer. An example of the implementation of displacement piles as soil reinforcement is presented in Figure 2. The technology is based on non-impact pile forming in the ground without spoil flowing to the ground surface. The specially designed drill pushes the soil sideways and compacts it at the edge of the pile sidewall during both immersion and withdrawal of the drill. A borehole is drilled in the first phase of pile formation. The drill is then raised while simultaneously feeding pressurised concrete. In this method, reinforcement can be introduced into the fresh concrete mix to increase the strength of the pile. Piles of this type can be made in all types of soil [62]. Buildings and engineering structures are indirectly founded on piles.

**Figure 3:**
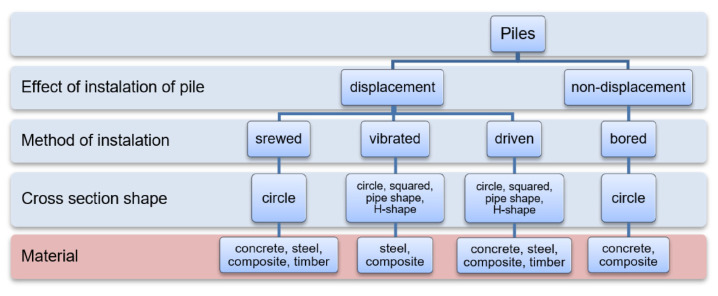
Pile classification.

Piles can be made in many technologies. They differ in the way they are made: on-site or prefabricated, the method of plunging, the shape, and the material. Figure 3 shows a general classification of piles. Piles made using different technologies, despite having the same geometry, have different bearing capacities. This is because the condition of the soil in the surroundings of the pile changes after it is made. The soil is compacted in displacement piles or loosened in the case of drilled piles. The pile resistance limits are also influenced by the roughness and quality of the pile/soil connection. In the case of piles made on-site, fresh concrete also binds soil grains at the pile-soil connection, improving the performance at the pile-soil contact surface [64].

The concrete mixture that is used in the construction of piles shall have good workability properties, high plasticity and self-compaction properties, and shall be resistant to segregation. Gravel aggregate concrete with a grain size of up to 16 mm and a consistency of S5 is most commonly used for soil-formed piles. The strength of the concrete depends on the designed bearing capacity of the pile, the ground and water conditions, as well as the execution technology. The concrete class for prefabricated piles with higher strength requirements shall be no less than C35/45. The high strength of the prefabricated pile allows for avoiding damage during driving. The strength of the concrete mix of piles cast on-site may be lower, provided that the required pile head strength and buckling resistance (in the case of weak soil or eccentric load) are met. The cement content of concrete mixes should not be less than 350 kg/m${}^{3}$. 3D printing systems do not offer soil reinforcement by making piles. The aim of this paper is to present the possibility of making concrete foundation piles using a 3D printer for concrete. The paper presents the tools and procedure for pile extrusion, describes the experiment for measuring the pile bearing capacity, and describes an example of a pile arrangement under the foundation while taking the obtained test results into account. The paper evaluates the solution presented.

## 2. Materials, Methods and Experiment Program

### 2.1. Foundation Pile—Assumptions

A concrete printer was used to make the piles, with a specially designed drill to make a hole in the soil and fill it with concrete mix. The pile construction technology is similar to soil displacement piles, called FDP—Full Displacement Pile or SDP—Soil Displacement Pile. Piles of this type are characterised by high bearing capacity, negligible spoilage and reduced concrete consumption [65]. The technology can be used to construct entire facilities that are made with 3D printing technology. In the displacement technology, when the soil is pushed by the immersed drill, the horizontal stress in the soil increases, which is reduced to the hydrostatic pressure of the concrete mixture after the soil is filled with concrete. The soil is pushed apart during drilling, which results in compaction and, therefore, improved strength parameters. The increased state of stress in relation to the original state also results in better cooperation of the pile with the soil.

The execution of piles using this technology also requires requirements to be met in relation to the displacement drill and the pile execution process. Because of the significant resistance during drilling, the design of the drill should be robust and the machine should initiate pressure during drilling. These pressures for naturally sized piles are around 100 kN. In the pile execution process, the drilling speed of the pile should be correlated with the rotational speed. The diameter of the pile will depend on the compressibility of the soil. In weaker soils, such as organic soils, plastic soils, or loose soils, the diameter of the pile will be larger than in rigid soils, such as compacted non-cohesive soils or compacted cohesive soils. When forming the pile shaft, it is important to properly correlate the mix discharge rate, mix pressure, and drill lifting speed. The correct maintenance of the correlation between these parameters is necessary to obtain constant pile parameters at its height, and it also prevents the formation of discontinuities in the pile caused, for example, by too low a pressure and speed of the mixture discharge in relation to the drill lifting speed.

The strength of the soil depends on the state of stress. Piles allow the better use of the soil’s bearing capacity due to the transfer of much of the load to deeper soil layers, which are in a state of higher geostatic stress. One of the main differences between piles and shallow foundations is their interaction characteristics with the soil. When piles are loaded to their ultimate capacity, the phenomenon of soil displacement to the surface that accompanies the loading of direct foundations is not observed. The ratio of the pile penetration (*H*) in the ground to its diameter (*D*) should be greater than 2.5 [66], but it is recommended that H/D ≥ 10. Taking the above technological and geometrical requirements into account, it was planned to execute piles with a drill with a diameter of 40 mm and a minimum soil penetration of 400 mm. Such a small pile diameter allows them to be classified as micropiles because *D* < 200 mm [67].

### 2.2. Soil

**Figure 4:**
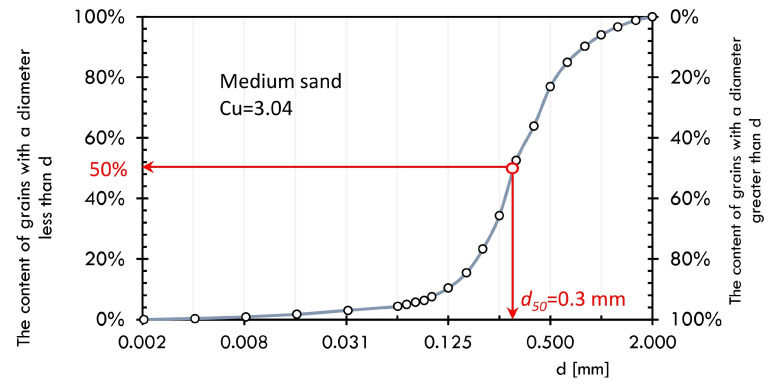
Grains distribution of sand (Cu—homogeneity index based on [70]).

Pile bearing capacity tests were conducted in non-cohesive soil with a grain size of 0–2 mm and a water content of 4.5% to 4.9%. Soil parameters along with grain size characteristics were determined based on Eurocode 7 requirements [68,69]. The minimum and maximum void ratios were determined with soil compacted densely or poured loosely into a metal mould respectively. Sand parameters are presented in the Table 1 and in the Figure 4. The soil used in the laboratory tests, due to its low water content and lack of cohesion, was characterised by good workability allowing for creating a soil environment of uniform compaction. In addition, the lack of cohesion made it possible to exclude its influence in load transfer.

### 2.3. The 3D Printer and Construction of a Drilling Rig

**Figure 5:**
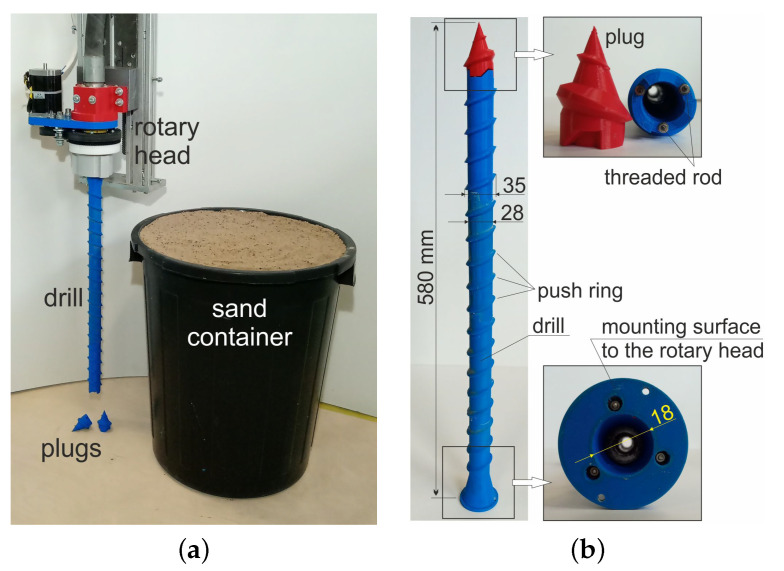
(**a**) Station for making foundation piles, (**b**) drill structure.

The tests were carried out on a system consisting of a concrete printer with Cartesian robot kinematics (3 DoF) with a working area of 1400 × 1200 mm and a height of 900 mm and a concrete mix pump. The printer’s working end was equipped with a rotating head with a mounted drill for making piles (Figure 5a). The structure of test version of drill consists of four tubular elements that were printed in polymeric material (printer: Zortrax M200, material: Z-PETG, layer thickness: 0.09 mm, infill density: 75%, pattern: linear) and then connected by a threaded rod (Figure 5b). On the outer surface of the drill, there are specially shaped rings whose purpose is to push and compact the soil during its operation. The concrete mix is extruded through the inner diameter of the drill. The drill is secured from the bottom with a conical pin (Figure 5b), which prevents soil from entering the drill during plunging and facilitates screwing it in. The plug is a stay-in-place component, it remains in the soil after the drill has been immersed. The piles were made in the ground placed in 500 mm high containers. Given the size of the printer workspace, it was assumed that the piles made as part of the experiment would be approximately 450 mm long and have a diameter in the range of 40–45 mm. In view of this, the ratio of the length of the pile to its diameter will be approximately 10.

**Figure 6:**
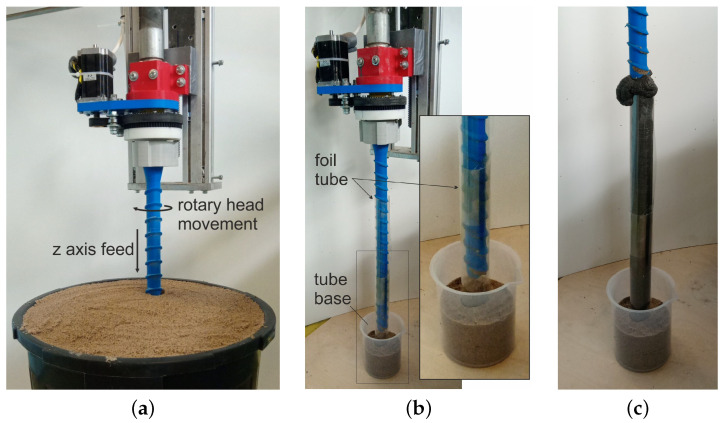
Tests for drill performance determination, (**a**) test of drill immersion, (**b**) test of pile extrusion in foil tube—starting position, (**c**) evaluation of tube filling with concrete (concrete extruded to tube).

Two tests were carried out prior to the execution of the target foundation piles. Test 1 consisted of driving the drill into the soil with assumed density parameters. The aim of the study was to determine the feed rate for the vertical axis of the printer and the rotation of the printing head, so as not to exceed the strength of the individual mechanisms. The figure (Figure 6a) shows the head with the drill during a plunge test in which the maximum feed rate was set at 100 mm/min. while the drill was rotated at 2.5 rpm. This test also examined the behaviour of the structure during drill removal. In this case, the resistance resulting from the interaction of the drill with the soil was very low. Accordingly, the drill feed rate that was used for the drill turning (i.e., extruding the concrete and forming the pile) was 800 mm/min. and the head speed was increased to 40 rpm.

Test 2 was designed to test the ability of the drill to pump the mix and determine the pump performance during pile forming with the assumed printer and rotary head movement parameters. The test rig consisted of a foil tube, embedded in a sand support, simulating the ground surrounding the drill (Figure 6b). Based on analytical calculations and visual assessment (Figure 6c) of the degree of filling of the foil tube with concrete, a mix extrusion rate of 1 L/min. was assumed.

### 2.4. Cement Mixes

For the planned experiments, a high-performance concrete mix was used, in which Portland cement CEM I 52.5R (70% of the binder), fly ash (20% of the binder), silica fume (10% of the binder), and natural fine aggregate of 0-2 mm fraction were used as the binding agents [19]. Table 2 presents the mix composition.

A trial mix extrusion test that was carried out revealed a technical problem. The mix was transported to the head with a 25 mm diameter hose and then in the drill the diameter is reduced to 18 mm over a length of approximately 580 mm. The rheological properties of the mix and the change in the diameter of the hose caused increased resistance to movement when the concrete was pumped and blocking of the material at the point of diameter reduction. It was decided to improve the pumpability of the mix by increasing the batch water and changing the w/c ratio from 0.345 to 0.495. This resulted in a more fluid mix, which made it possible to form piles in the soil with a given drill diameter. As a result of the modification, the compound no longer has the right rheological properties to be suitable for 3D printing. However, the piles are made in a soil surrounding environment so there is no requirement for the concrete to have adequate buildability. The proportions of individual mineral binders, the binder/aggregate ratio, and the superplasticizer/binder ratio remained the same. Table 2 and Table 3 show the composition and mass proportions of the components of the base concrete (B829/W200—BASE) and the concrete for piles with increased water content (B766/W265—PALE), respectively.

**Figure 7:**
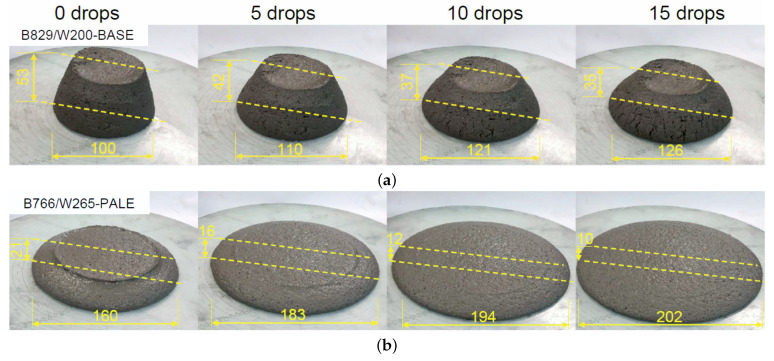
Testing the consistency of 3D mortars using the slump-flow table method: (**a**) initial mortar B829/W200—BASE [19], (**b**) modified mortar B766/W265—PALE.

**Figure 8:**
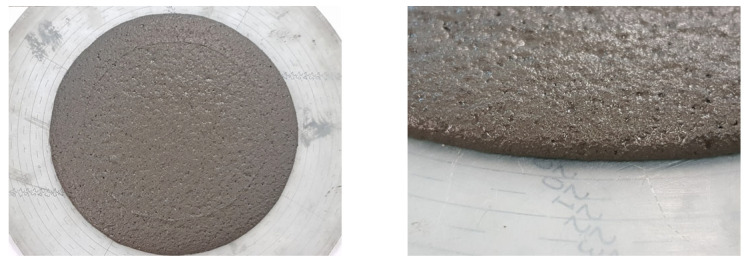
Evaluation of visual stability index—VSI0 for the B766/W265—PALE mix.

A comparative consistency test of the two mixes using a slump flow table after 0, 5, 10, and 15 compaction impacts was carried out (Figure 7). Increasing the water/cement ratio to 0.495 increased the slump diameter of the mixture, but it did not cause bleeding or segregation of the mixture. Figure 7 shows the changes in spread and cone drop as a function of table impact for both of the mixes. The visual stability index (VSI) was assessed as VSI0 (Figure 8).

**Figure 9:**
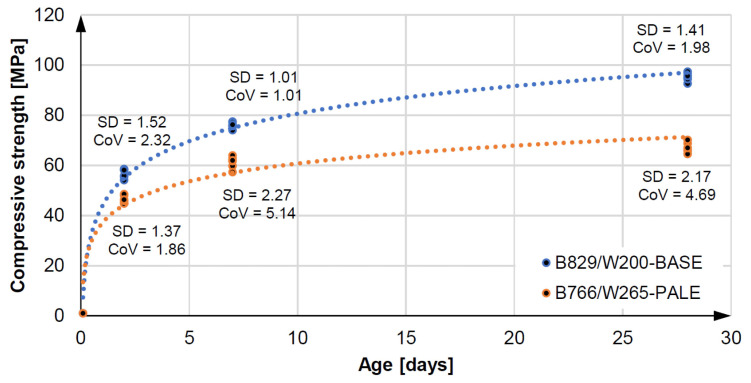
Compresive strenght and statistic parameters of 3D concretes.

**Figure 10:**
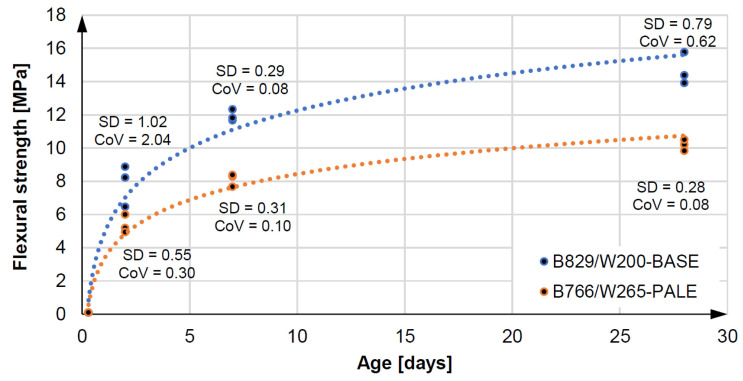
Fluxural strenght and statistic parameters of 3D concretes.

Increasing the batch water reduces the strength of the original Equationtion concrete, while it still retains very good mechanical performance. Flexural and compressive strength tests were carried out on three samples of 40 × 40 × 160 mm and six samples of 40 × 40 × 40 mm each after two, seven, and 28 days according to EN 196-1 [71]. Figure 9 and Figure 10 present the results of compressive and bending strength tests for the specimens, respectively. The B766/W265—PALE pail mix has an average compressive strength of 67.01 MPa and an average flexural strength of 10.58 MPa after 28 days. This means that the reduction in flexural and compressive strength after 28 days is within 30% of the B829/W200-BASE base mix. In practice, the concrete of maximum class C30/37 or C35/45 is usually used for on-site piles [68]. Mix preparation, pile construction, and curing in the ground were carried out at an ambient temperature of 20 ${}^{\circ }$C ± 2 ${}^{\circ }$C and an air humidity of 60% (±5%).

The total shrinkage of 3D concrete was tested by the Graf–Kaufman [72] method on three samples 50×50×250 mm in the range from one to 28 days. The modulus of elasticity was tested after 24 h and 28 days according to European Standard [73]. Table 4 presents the results. Fresh mix density was 2250 kg/m${}^{3}$, while, after 28 days, hardened concrete was 2200 kg/m${}^{3}$.

### 2.5. Foundation Pile Extrusion Procedure

**Figure 11:**
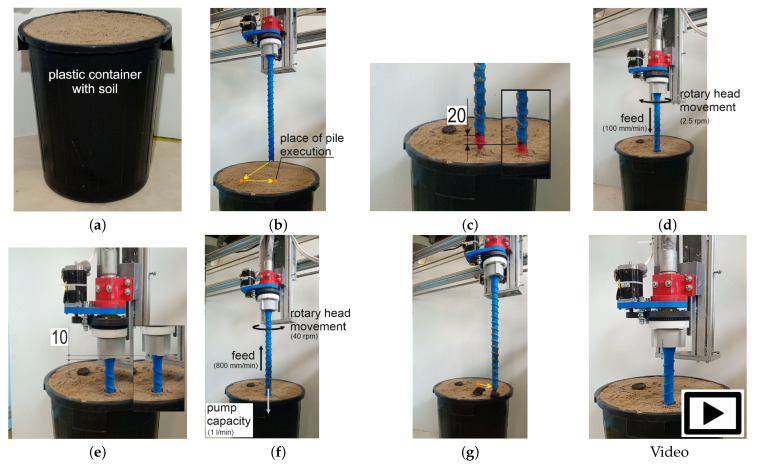
Stages of performing a foundation pile: (**a**) Stage 1—Preparing the soil in the container, (**b**) Stage 2—positioning the printer over the place of performing the pile, (**c**) Stage 3—assembling the plug and pressing it to the soil, (**d**) Stage 4—immersing the drill, (**e**) Stage 5—disconnecting the drill and the plug, (**f**) Stage 6—forming the foundation pile, and (**g**) Stage 7—finishing the work. Video: See Supplementary Materials.

The process of making foundation piles was divided into five stages:

- Stage 1: preparing the containers with sufficiently compacted soil. Note: the next day after the piles were made, soil samples were taken from the containers to calculate their degree of compaction (Figure 11a).
- Stage 2: positioning the printer above the foundation pile construction site (Figure 11b).
- Stage 3: assembly of the tapered plug. The plug was assembled by hand. The *Z*-axis of the printer was then lowered by 20 mm to seat the plug in the drill socket by pressing it into the soil (Figure 11c).
- Stage 4: immersing the drill in the soil using a rotary head (without pumping concrete). The drill is driven into the soil to a depth of 460 mm with a vertical axis feed rate of 100 mm/min. and a head rotation speed of 2.5 rpm (Figure 11d).
- Stage 5: initial raising of the drill. The drill is raised to a height of 10 mm. The drill and the plug are disconnected to prevent the plug from jamming in the drill (Figure 11e).
- Stage 6: forming the foundation pile. The drill is removed while the cement mix is extruded. With a vertical axis feed speed of 800 mm/min. and head speed 40 rpm, with a pump capacity of 1 L/min (Figure 11f).
- Stage 7: completion of the pile pumping and departure of the printer (Figure 11g).

### 2.6. Strength/Load Capacity Testing of Piles

**Figure 12:**
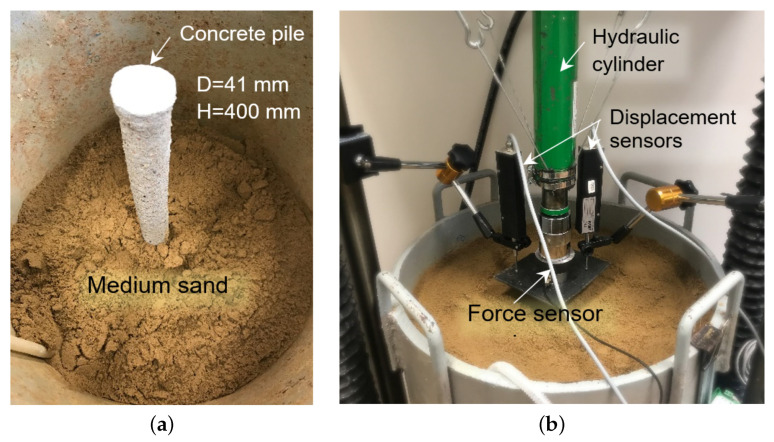
Test rig for pile bearing capacity (**a**) compaction of soil around the pile, (**b**) foundation pile prepared for testing.

The pile test rig consisted of a 48.5 cm diameter, 60 cm high chamber. The soil was placed in the chamber in layers and compacted dynamically with a plate. Each layer was compacted with the same compaction energy to achieve uniform compaction. The pile was placed in the chamber while the chamber was being filled with soil (Figure 12a). Compaction was checked by taking intact samples using a steel cylinder [69,70]. This sample was taken by inserting a steel cylinder and then a larger diameter casing the pipe to act as a sample separator from the surrounding soil. Two additional samples were taken from each sample for water content measurement. These data enabled the determination of the natural porosity index, which was used to calculate the density ratio of the soil. The measurement of the total mass and volume of soil in the chamber was also used to verify the soil compaction.

A hydraulic cylinder was used to load the pile with an axial force that caused it to settle into the soil (Figure 12b). During the test, the pile settlement was recorded, which stabilised when the load was kept constant. The transition to the next load step took place when the condition of stabilisation of settlement of no more than 0.02 mm/min. was met. The force was measured using strain gauge transducers that were placed in the head. Whereas the pile movement was measured using two optoelectronic displacement sensors with a measurement range of 0–50 mm and an accuracy of 0.005 mm. The measured values were recorded at a frequency of 1 Hz. In the conducted study, the pile bearing capacity was defined by two criteria: load at which uncontrolled pile settlement was observed (this is characteristic of a load close to the limit bearing capacity) [66] or the force at which the pile settlement exceeded the value of 20 mm.

The test bed that was prepared in this way may only be used for one test pile load. As indicated by the previous studies [66,67,74,75], performing a pile test load to a load value close to the ultimate bearing capacity results in a critical condition at which the soil porosity index changes to reach the so-called steady state line and critical porosity [76,77,78,79]. As a result, the soil environment becomes heterogeneous. In order to perform all of the tests in a homogeneous soil environment, the rig was built for each test separately while applying a different compaction energy. In this way, the piles were tested in soil with varying degrees of compaction from $D_{R}$ = 34% (loose soil) to $D_{R}$ = 70% (compacted soil). The testing of the piles was performed after 28 days of their curing in the soil.

### 2.7. Measurements of Pile Geometries

**Figure 13:**
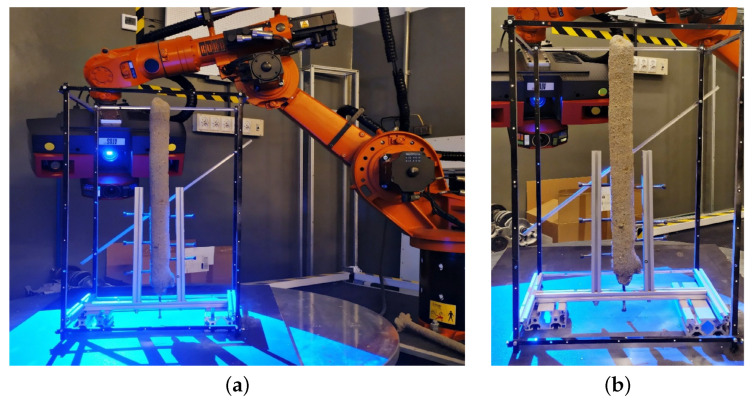
Optical scanning system (**a**) during the measurement of a foundation pile placed in the measuring frame, (**b**) a measuring fixture holding the pile still during the measurement.

For the calculation of the bearing capacity of a foundation pile, the surface area of the pile sidewall must be determined. The necessary geometrical parameters of the component under test were measured using a numerical model. This model was obtained by scanning the pile using the Atos III Triple Scan optical scanner (GOM GmbH, Braunschweig, Germany) that was installed on an industrial robot with an integrated rotary table (Figure 13a).

Equipped with a structured light projector and dual-camera optics, the head enables the shape and geometry of the measured object to be reconstructed by scanning the surface. The matching of successive scans is carried out using reference points (so-called markers) that were placed in the vicinity of the object under examination. The marker technology makes it possible to omit the low accuracy of the industrial robot, which is only a manipulator, and to achieve high accuracy in the measurement performed, since the measurement is not based in the robot’s coordinate system [80].

A frame with applied markers was used to scan the foundation piles, and the scanned object was fixed in place with screws being placed in the fixture (Figure 13b). The measurement, after the preparation of successive movements of the head and rotary table, was performed in an automatic cycle, allowing for the best repeatability of scanning conditions for each sample tested. The repeatable accuracy of the reproduction of the three-dimensional geometry of an object is up to 4 μm; however, this is the highest achievable accuracy value, for which many strictly defined conditions must be met. Therefore, the average accuracy of the scans performed is about 50 μm, as confirmed by scanner accuracy studies under different environmental conditions [81,82].

### 2.8. Experimental Design—Summary

The main assumptions for conducting the soil displacement pile pumping experiment are as follows:

- Soil: non-cohesive soil, soil compaction 30–70%, 0–2 mm fraction, water content: 4.5–4.9%.
- Respectable dimensions of piles: lengths: 450 mm, external diameters: 40–45 mm.
- Number of piles: 7 pcs.
- Pile printing parameters: feed rate of the vertical axis: 800 mm/min, head rotational speed: 40 rpm, pump capacity: 1 L/min.
- Mix: B766/W265—PALE
- Pile strength testing: after 28 days of curing in the soil.

## 3. Experimental Results and Discussion

### 3.1. Foundation Piles

**Figure 14:**
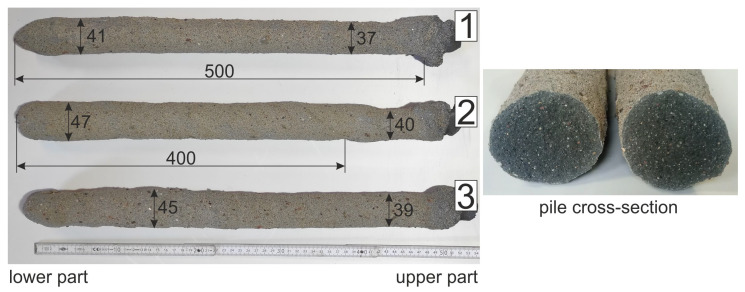
Dimensions of pumped displacement piles.

Figure 14 shows the dimensions of three example piles and the cross-section of the pile after breaking. The resulting piles are of uniform length, but the diameter of the pile along its length varies. The cross-section of the pile shows that the material is homogeneous without air voids. When testing the load bearing capacity of the piles, they will be placed in the soil at a depth of 400 mm.

**Figure 15:**
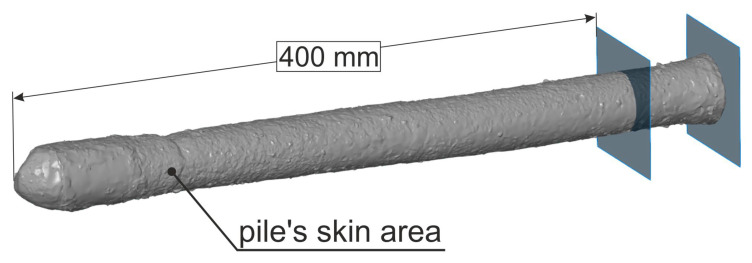
Pile’s skin area calculated based on the scan.

On the basis of a scan of the completed piles, its sidewall areas were calculated over a length of up to 400 mm (Figure 15). The average sidewall area for the three sample piles was 63,950 mm${}^{2}$ and the average diameter was 41.5 mm.

### 3.2. Bearing Capacity of Foundation Piles

The test of the pile bearing capacity consisted in carrying out a static test load, with fixed load steps being assumed. The result of the test is a load-settlement function $N_{2}\left(s\right)$ describing the stabilisation of pile settlement at different load steps. The course of this function depends, among other things, on: the geometry of the pile, the ground conditions, and the way resistance is formed with pile settlement. The points obtained from the test can be approximated by the curve that was proposed by Meyer and Kowalow in the form of Equation (1) presented in [66,83,84].

(1) $$N_{2}\left(s\right)=N_{2,gr}\left[1-{\left(1+\frac{s\kappa_{2}}{C_{2}N_{2,gr}}\right)}^{-1/\kappa_{2}}\right]$$

where: s—settlement of the head of the pile, mm; $C_{2}$—settlement constant, mm/kN;

$N_{2,gr}$—ultimate pile load capacity when uncontrolled settlement of the head of the pile is observed, kN; $\kappa_{2}$—dimensionless parameter of settlement curve; and, $N_{2}$—axial load of the pile, kN.

The transformation of Equation (1) provides the relationship (2) describing the value of pile settlement as a function of load.

(2) $$s\left(N_{2}\right)=\frac{C_{2}N_{2,gr}}{\kappa_{2}}\left[{\left(1-\frac{N_{2}}{N_{2,gr}}\right)}^{-\kappa_{2}}-1\right]$$

**Figure 16:**
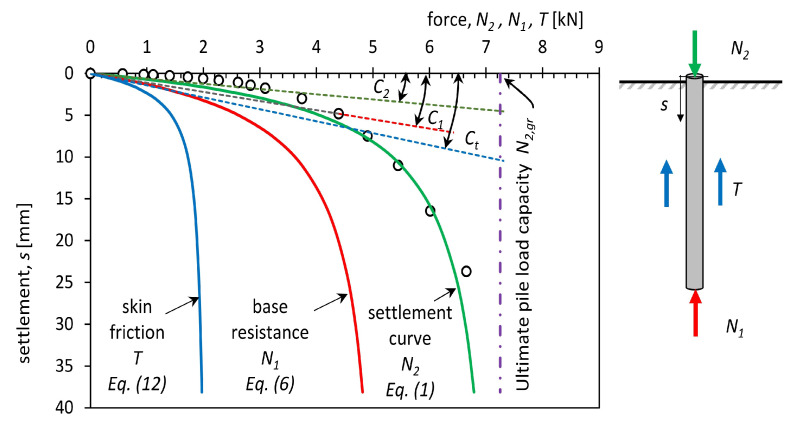
Measured settlement versus applied load—(dots) and calculated curves: settlement approximation curve (green line); base resistance curve (red line) and skin friction curve (blue line) of pile no 4.

The load at which pile settlement reaches significant values (50–200 mm) is characteristic of the pile–soil cooperation. In mathematical terms, it is a vertical asymptote defined by the limit value of the force $N_{2,gr}$, which must not be exceeded (Figure 16) to prevent uncontrolled pile settlement. Another parameter of the pile settlement curve is the constant $C_{2}$, which determines the slope of the diagonal asymptote at the origin of the coordinate system (Figure 16). This parameter determines the elastic character of the pile’s cooperation with the soil at low loads.

On the basis of laboratory tests of static test loads on piles with reference geometries $D_{Ref}$ = 0.041 m and $H_{Ref}$ = 0.4 m, Equations (3)–(5) were derived that determine parameters $N_{2,gr}$, $\kappa_{2}$, $C_{2}$, which depend on the change in soil compaction $D_{R}$:

(3) $$N_{2,gr}=48.753D_{R}^{3.1881}$$

(4) $$\kappa_{2}=1.2327exp\left(-\frac{0.306}{D_{R}}\right)$$

(5) $$C_{2}=0.1162D_{R}^{-2.804}$$

The bearing capacity of a foundation pile can be decomposed into a resistance force that results from the pile skin friction against the soil and a resistance force resulting from the pressure of the pile base on the soil (Figure 16). The change in pile base resistance as a function of settlement is described by Equation (6) and Equations (7)–(9), being verified by experimental laboratory and field tests [74].

(6) $$N_{1}\left(s\right)=N_{1,gr}\left[1-{\left(1+\frac{s\kappa_{1}}{C_{1}N_{1,gr}}\right)}^{-1/\kappa_{1}}\right]$$

where:

(7) $$C_{1}=C_{2}{\left(0.467\kappa_{2}+1\right)}^{2}$$

(8) $$\kappa_{1}=0.833\kappa_{2}$$

(9) $$N_{1,gr}=C_{2}N_{2,gr}/C_{1}\left[1+0.1435{\left(H/D\right)}^{1/3}\kappa_{2}^{0.5}\right]$$

where $C_{1}$—settlement constant due to pile base resistance, mm/kN; $N_{1,gr}$—ultimate base resistance, kN; $\kappa_{2}$—dimensionless parameter of pile base resistance curve; *D*—diameter of the pile of any length, m; and, *H*—depth of the pile in the ground, m.

The skin resistance *T* can then be calculated as the difference of the force applied at the pile head $N_{2}$ and the mobilised resistance under the pile base $N_{1}$ calculated from Equations (6)–(9) (the balance of forces is schematically shown in Figure 16):

(10) $$N_{2}\left(s\right)=N_{1}\left(s\right)+T\left(s\right)$$

(11) $$T\left(s\right)=N_{2}\left(s\right)-N_{1}\left(s\right)$$

(12) $$T\left(s\right)=N_{2,gr}\left[1-{\left(1+\frac{s\kappa_{2}}{C_{2}N_{2,gr}}\right)}^{\frac{-1}{\kappa_{2}}}\right]-N_{1,gr}\left[1-{\left(1+\frac{s\kappa_{1}}{C_{1}N_{1,gr}}\right)}^{\frac{-1}{\kappa_{1}}}\right]$$

Using the procedure that is outlined above, parameters $N_{2,gr}$, $\kappa_{2}$, $C_{2}$ were determined for all of the piles tested. Table 5 shows the parameters obtained.

**Figure 17:**
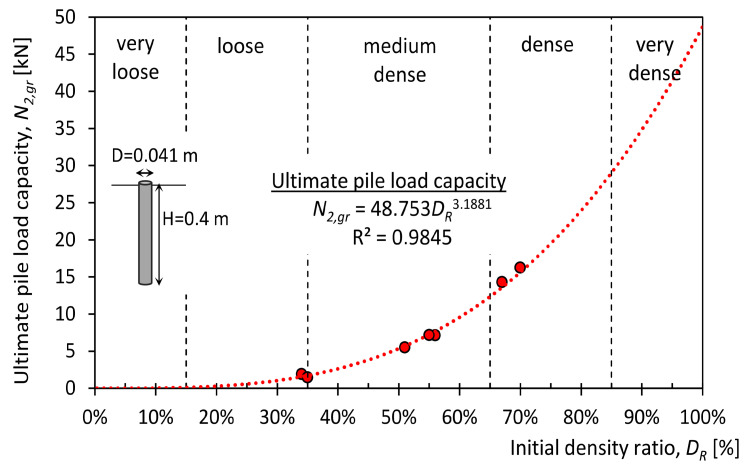
Pile load capacity versus density ratio of soil (experimental values—red dots, approximation model—red dashed line).

Figure 17 shows the limit load capacities that were obtained from approximation with Equation (3) for different soil compaction (red points) and their approximation with Equation (1) (red dashed line).

## 4. Structural Design of a Building with Concrete Piles

**Figure 18:**
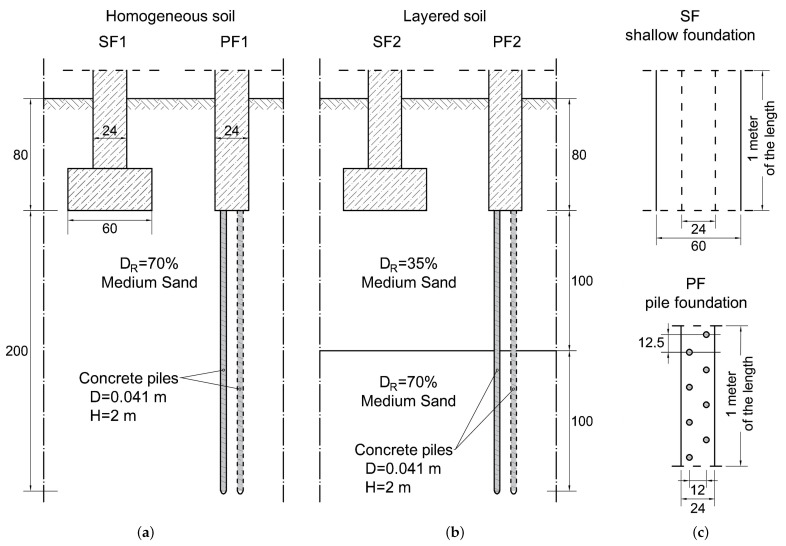
Schemes of foundations and geotechnical profiles: (**a**) Shallow (SF1) and pile (PF1) foundation in homogeneous soil, (**b**) Shallow (SF2) and pile (PF2) foundation in layered soil, annd (**c**) top view of 1 m length of the both foundations. Dimensions in centimeters.

Four structures were modelled for the foundation of a building in order to test the effectiveness of the piles made using a 3D printing system. Models SF1 (Figure 18a) and SF2 (Figure 18b) show a typical shallow foundation (SF) made on a footing 0.6 m wide and founded 0.8 m below ground level. Models PF1 (Figure 18b) and PF2 (Figure 18b) simulate a piled foundation (PF) consisting of a narrow 0.24 m wide cap connected to piles. The piles were assumed to be 2 m long and 4.1 cm in diameter, with a regular, edge-to-edge spacing of no more than eight piles per 1 m. The foundations in SF1 and PF1 are made on homogeneous soil with a compaction of 70%. The SF2 and PF2 models, on the other hand, assumed that the soil beneath the foundations consisted of two layers with 35% and 70% compaction. Table 6 shows the foundation assumptions, while Table 7 shows the soil parameters that were adopted in the simulations.

The bearing capacity and settlement of the direct foundation were calculated using the procedure that was described in Eurocode 7 [68,85]. When calculating the bearing capacity of a foundation on piles, the resistance of the pile side wall up to a depth of 1 m below the strip foundation was ignored due to the simultaneous settlement of soil and pile in this zone [86]. The load-bearing capacity of piles is achieved by pile side resistance mobilised at depths beyond the influence zone of the direct foundation. The analysis was carried out based on analytical Equations to show the stages of calculation, but it can also be improved by numerical modeling [87].

Equations (1), (6), and (12) described the bearing capacity of the foundation piles adopted in the simulation. The parameters of these functions were estimated on the basis of [88,89] taking the geometry of the piles and the results of the load capacity of experimentally tested piles of reference diameter $D_{ref}$ = 0.041 m and reference length $H_{ref}$ = 0.4 m into account.

For small pile loads, i.e., $N_{2}\left(s\right)≅0$ settlement is determined by the diagonal asymptote that is defined by the factor $C_{2}$ because $s=C_{2}N_{2}$ according to Figure 16. This asymptote is the result of the simultaneous interaction of the pile skin and base expressed as the inverse of the Winkler coefficient, Equation (13), relating the resistance components of the pile skin and base.

(13) $$\frac{1}{C_{2}}=\frac{1}{C_{1}}+\frac{1}{C_{t}}$$

where: $C_{t}$—constant of an oblique asymptote referring to skin friction, mm/kN.

The susceptibility of the pile base to settlement in the range of near-linear deformations can be calculated from the pile diameter and oedometer modulus of the soil at the level of the pile base according to the general Equation (14) [89].

(14) $$C_{1}=\frac{1}{\pi q_{b}\alpha D}$$

For the reference pile, this susceptibility is described by the Equation (15).

(15) $$C_{1,ref}=\frac{1}{\pi q_{b}\alpha D_{ref}}$$

where: $q_{b}\alpha =E_{p}$—oedometer modulus of soil at the base level, MPa; $q_{b}$—cone resistance of CPT (Cone Penetration Test), MPa; $C_{1,ref}$—calculated parameter of settlement curve of the referenced pile, mm/kN; and, $D_{ref}$—diameter of referenced pile 0.041 m.

After the transformation of Equations (14) and (15), the function describing the susceptibility of the pile base with diameter *D* is determined on the basis of the susceptibility and diameter of the referenced pile.

(16) $$C_{1}=C_{1,ref}\frac{D_{ref}}{D}$$

The susceptibility of the pile skin to settlement in terms of linear deformation can be calculated on the basis of the diameter and length of the pile and the modulus of elasticity of the soil according to Equation (17) [89].

(17) $$C_{t}=\frac{G}{\pi l}\frac{1}{DH}$$

where: *G*—bulk modulus of soil, MPa.

For the referenced pile, the Equation (18) described the susceptibility of the pile skin.

(18) $$C_{t,ref}=\frac{G}{\pi l}\frac{1}{D_{ref}H_{ref}}$$

where: $C_{t,ref}$—calculated parameter of skin friction mobilizing curve of the referenced pile, mm/kN; *l*—length of soil deflection surrounding the pile, m.

Equations (17) and (18) were used to determine the function describing the susceptibility of a pile skin of any diameter *D* and length *H* on the basis of the susceptibility and geometry of the reference pile.

(19) $$C_{t}=C_{t,ref}\frac{D_{ref}}{D}\frac{H_{ref}}{H}$$

The ultimate bearing capacity of the pile base can be calculated as the product of the unit limiting resistance of the CPT probe cone and the area of the pile base. Usually, in practice, the ultimate base resistance should also consider the reduction factor, but this factor is only applicable for piles for which steady penetration is not reached. The analysed piles in the paper were analysed to the failure criterium that was described by uncontrolled settlement typical for steady penetration. In this way, the unit base resistance can be compared with cone resistance of CPT soundings [90]. Equation (20) describes the bearing capacity $N_{1,gr}$ for a pile of any diameter *D*, while Equation (21) describes this bearing capacity for a reference pile.

(20) $$N_{1,gr}=\frac{\pi q_{b}D^{2}}{4}$$

(21) $$N_{1,gr,ref}=\frac{\pi q_{b}D_{ref}^{2}}{4}$$

In turn, the ultimate bearing capacity of the pile base as a function of the reference pile parameters is as follows:

(22) $$N_{1,gr}=N_{1,gr,ref}{\left(\frac{D}{D_{ref}}\right)}^{2}$$

The maximum skin resistance can be calculated as the product of the limit shear strength and the shaft area of the pile [89]. Equation (23) describes the maximum skin friction for a pile of any geometry, Equation (24) describes the same resistance for a pile of reference geometry, while Equation (25) defines the maximum skin resistance for a pile of any diameter and length with respect to the reference pile.

(23) $$T_{max}=\tau_{f}\pi DH$$

(24) $$T_{max,ref}=\tau_{f}\pi D_{ref}H_{ref}$$

(25) $$T_{max}=T_{max,ref}\frac{D}{D_{ref}}\frac{H}{H_{ref}}$$

After transforming Equation (7), (26) is obtained.

(26) $$\kappa_{2}=2.14\left(\sqrt{\frac{C_{1}}{C_{2}}}-1\right)$$

**Figure 19:**
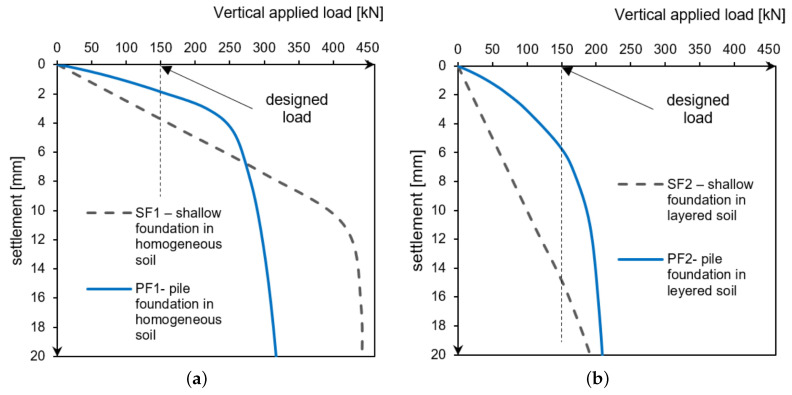
The load–foundation settlement relationship (**a**) for homogeneous soil, (**b**) for layered soil (with a weaker layer located directly below the direct foundation).

Equations (3)–(5) obtained from the static test loads and Equations (7)–(9), (18), (22), (24), and (26) made it possible to calculate the load capacity of piles of different lengths. In the following section, two types of foundations are analysed. The reference point in the analysis performed is the assumed design load value of 150 kN/mb. Table 8 and Figure 19 show the results of the calculations.

Foundation in homogeneous soil: SF1, PF1. In a homogeneous subsoil with a compaction of 70%, the abandonment of the shallow foundation in favour of piles resulted in a reduction of the ultimate bearing capacity. Nevertheless, the application has also brought positive aspects. In the design load range of 150 kN/m, the pile foundation is stiffer than the standard direct foundation, as the settlement at the design load was about 2 mm for piles and 4 mm for shallow foundations (Figure 19 and Table 8).

Foundation in layered soil: SF2, PF2. In a layered subsoil with shallow weak layers, the use of piles instead of wide shallow foundation has proved to be advantageous. The bearing capacity of both the direct and pile foundations proved to be comparable. However, the proposed pile foundation is less susceptible to settlement, as the bearing capacity results from the strength of soils lying deeper than the zone of influence of the direct foundation. The analysed building founded on piled foundations will reach the settlement of approximately 5 mm with the design load, while the direct foundation that is compared in this analysis will reach as much as 15 mm (Figure 19 and Table 8).

Table 9 shows a comparison of the consumption of materials that are needed for a foundation in the form of a shallow foundation and a pile foundation. All of the materials are calculated per 1 m of foundation. The saving (last column of Table 9) was calculated according to Equation (27):

(27) $$Q_{f}=\frac{Z_{sf}-Z_{pf}}{Z_{sf}}\times 100\%$$

where: $Z_{sf}$—the consumption of materials needed to make the piles using the 3D printing robot; $Z_{pf}$—consumption of materials needed for a traditional shallow foundation; and, $Q_{f}$—savings in material consumption expressed as a percentage.

Based on Table 9, in the case of traditional shallow foundations, the concrete consumption is as much as 53.0% higher than in the case of piles that formed using a 3D printer. This results in a significant reduction in printing costs and, due to the reduced use of cement and other reactive components, has a positive impact on the environment [38,91]. In addition, a smaller amount of concrete directly reduces the need for formwork, which is a significant cost when erecting building structures [92,93]. An additional advantage of making piles with the help of a 3D printing robot is the reduction of earthworks by as much as 68.3% (Table 9), which reduces the costs and speeds up the entire construction process. Assuming that the entire facility is built using incremental technology, we do not significantly increase the costs that are associated with the deployment of equipment for this purpose. An additional advantage of using a 3D printer to form piles in the ground is the reduction in the number of people that are employed to make such piles, which is also related to the cost of performing this type of structure. In conclusion, the use of the proposed technology, apart from saving time and money, is also beneficial for the environment.

## 5. Conclusions

The results of the experiments confirmed the technical feasibility of constructing piles using a 3D printer and the usefulness of pile bearing capacity tests for designing soil reinforcement while using these elements. The facilities and equipment used to pump the piles into the soil and the procedure for doing so are also described. The experimental studies and simulations carried out have led to the following conclusions:

**Mix**

- To improve pumpability, the mix with an increased amount of water was used during the pile construction process (w/c from 0.345 to 0.495).
- The increased amount of batch water resulted in a decrease in the compressive and flexural strength of the mix. The decrease in strength after 28 days of curing was within 30% of the base mix strength. The obtained compressive strength of the modified concrete is about 67 MPa and it meets the strength requirements that a pile of the proposed geometry must have [70].
- The modification of the amount of water in the mix can be very easily implemented and automated in the printing mix preparation system. This makes it possible to use the same material base used to make, for example, walls with incremental technology.

**Piles**

- No problems with stability, buckling, or failure of the printed piles were observed during the pile capacity tests. The pile foundation was characterised by both the continuity and repeatability of the geometric parameters.
- The results that were obtained from the pile bearing capacity test allowed for the approximation/estimation of the bearing capacity for piles with a different geometry.

**Simulation**

- The printed displacement piles, despite their lower bearing capacity, showed lower settlements when compared to the analysed shallow foundation.
- On the basis of the simulations carried out, it can be concluded that foundations consisting of a small cap and piles made with the technology (proposed in this work) can successfully replace a standard shallow foundation on shallow foundations.
- The pile foundation showed less sensitivity to the presence of weaker subsoil layers immediately below the foundation. Through the piles, the load is transferred to deeper soil layers with a higher bearing capacity.
- A building founded on piles is characterised by a small and uniform settlement due to the high stiffness of the foundation that results from the larger area of soil incorporated into the cooperation with the foundation.
- Using 3D printed piles as the foundation of a lightweight building can be an alternative to a standard direct foundation.

**Technology: device, process**

- The length of the piles is limited by the height of the printer. The drill that was used in the research was a test version made of polymeric material, which allowed for the construction of piles with a maximum length of 0.5 m. The authors assume that the minimum length of this type of pile should be 3 m and the diameter should vary between 5–10 cm. For this purpose, the drill should be longer and made of a material with higher strength. However, the geometry of the piles and their distribution in the soil primarily depend on the geotechnical conditions and the load that is transferred from the structure to the substrate.
- The construction of a printer equipped with a pile-driving head must be able to cope with the higher loads that result from drilling in the ground.

**Costs**

- Because of a reduction in the amount of earthworks, the amount of material used, and the amount of formwork (Table 9), the cost of founding a building on printed piles will be significantly lower than in the case of a building founded on a traditional shallow foundation.
- The use of pile foundations saves up to approx. 70–75% of concrete in comparison to a standard shallow foundation.

## Figures and Tables

**Table 1:** Parameters of medium sand.

$e_{\max}$	$e_{\min}$	w, %	$d_{50}$, mm
0.776	0.452	4.5–4.9	0.3

where: *e_max_*—maximum soil porosity index; *e_min_*—minimum soil porosity index, *w*—soil water content, %; *d_50_*—substitute average of grains which together with the smaller ones account for 50%, mm.

**Table 2:** Composition of concrete mixes, unit kg/m${}^{3}$.

Materials	CEM I 52.5R	Fly Ash	Silica Fume	SP	Water	Sand 0-2
B829/W200 BASE	580	166	83	12	200	1290
B766/W265 PALE	536	153	77	11	265	1191

**Table 3:** Mass ratios of composition of concrete mixes.

Mass Ratios	FA/C	SF/C	W/C	W/B	SP/B	B/S
B829/W200 BASE	0.286	0.143	0.345	0.241	0.014	0.643
B766/W265 PALE	0.286	0.143	0.495	0.346	0.014	0.643

where: FA/C—fly ash/cement, SF/C—silica fume/cement, W/C—water/cement, W/B—water/binder, SP/B—superplasticizer/binder, B/S—binder/sand.

**Table 4:** Results of total shrinkage and elastic modulus for examined concrete.

Time, days	Total Shrinkage, μm/m	CoV, %	Elastic Moduls, GPa	CoV, %
1	0	0.00	8.18	2.92
2	320	5.52	-	-
5	718	1.60	-	-
7	835	1.38	-	-
14	968	1.19	-	-
28	1055	1.05	33.87	3.15

where: CoV—Coefficient of Variation.

**Table 5:** Parameters of settlement curves and resistances mobilization curves of the investigated piles (based on Equations (3)–(5)).

No.	1	2	3	4	5	6	7
$D_{R}$, %	34	35	51	55	56	67	70
$N_{2,gr}$, kN	1.56	1.72	5.78	7.25	7.68	13.54	15.64
$\kappa_{R}$, -	0.501	0.514	0.607	0.707	0.714	0.761	0.796
$C_{2}$, mm/kN	2.39	2.21	0.72	0.62	0.59	0.42	0.32

**Table 6:** The geometry of the analysed shallow SF and pile PF foundations.

Parameter		Foundation Type	
		SF—Shallow Foundation	PF—Pile Foundation
Shallow foundation width	*B*, m	0.6	0.24
Length of piles 3D	*H*, m	-	2.0
Shallow foundation length	*L*, m	10	10
Depth ratio	*D*, m	0.8	0.8

**Table 7:** Geotechnical parameters of soils which were asummed in analysis.

Geotechnical Parameter		Soil Type	
		Loose Medium Sand	Dense Medium Sand
Bulk density	γ, kN/m${}^{3}$	16	18
Angle of internal friction	φ, deg	35	30
Oedometer modulus	$E_{oed}$, MPa	60	15
Density ratio	$D_{R}$, %	35	70

**Table 8:** The results of shallow and pile foundation calculation in homogenous/layered soil described in Figure 18.

		Scheme 1: Homogeneous Soil Dense Sand		Scheme 2: Layered Soil Loose and Dense Sand	
		SF1	PF1	SF2	PF2
Load capacity, kN	Shallow foundation [70]	439.96	139.45	202.25	66.26
	Piles (Equations (1)–(8) and (14)–(26))	0	220.61	0	220.61
	Ultimate load capacity:	439.96	360.06	202.25	286.87
Settlement at design load 150 kN, mm		3.75	2.13	15.00	4.82

where: SF1—shallow foundation in homogeneous soil, PF1—pile foundation in homogeneous soil, SF2—shallow foundation in layered soil, PF2—pile foundation in layered soil.

**Table 9:** Comparison of materials needed for performing foundation.

No.	Element	Piles $Z_{\mathrm{sf}}$	Shallow Foundation $Z_{\mathrm{pf}}$	Savings $O_{f}$, %
1.	Concrete consumption, m${}^{3}$/m	0.14	0.30	53.0
2.	Earthworks, m${}^{3}$/m	0.32	1.01	68.3
3.	Consumption of reinforcing steel, kg/m	0	5.24	100
4.	Formwork, m${}^{2}$/m	0	1.60	100

## Data Availability

The data presented in this study are available within the article. They are also available on request from the corresponding author.

## Supplementary Materials

The following are available online at https://www.mdpi.com/1996-1944/14/10/2545/s1.
